# Analysis of Comorbidities, Clinical Outcomes, and Parathyroidectomy in Adults With Primary Hyperparathyroidism

**DOI:** 10.1001/jamanetworkopen.2022.15396

**Published:** 2022-06-03

**Authors:** Kristian F. Axelsson, Märit Wallander, Helena Johansson, Nicholas C. Harvey, Liesbeth Vandenput, Eugene McCloskey, Enwu Liu, John A. Kanis, Henrik Litsne, Mattias Lorentzon

**Affiliations:** 1 Region Västra Götaland, Närhälsan Norrmalm, Health Centre, Skövde, Sweden; 2 Sahlgrenska Osteoporosis Centre, Department of Internal Medicine and Clinical Nutrition, Institute of Medicine, University of Gothenburg, Gothenburg, Sweden; 3 Mary MacKillop Institute for Health Research, Australian Catholic University, Melbourne, Victoria, Australia; 4 Medical Research Council (MRC) Lifecourse Epidemiology Centre, University of Southampton, Southampton, United Kingdom; 5 National Institute for Health Research Southampton Biomedical Research Centre, University of Southampton and University Hospital Southampton National Health Service Foundation Trust, Southampton, United Kingdom; 6 Centre for Metabolic Bone Diseases, University of Sheffield Medical School, Sheffield, United Kingdom; 7 MRC Versus Arthritis Centre for Integrated Research in Musculoskeletal Ageing, Mellanby Centre for Musculoskeletal Research, University of Sheffield, Sheffield, United Kingdom; 8 Region Västra Götaland, Department of Geriatric Medicine, Sahlgrenska University Hospital, Mölndal, Sweden

## Abstract

**Importance:**

Patients with primary hyperparathyroidism (pHPT) appear to have an increased risk of fractures and other comorbidities, such as cardiovascular disease, although results from previous studies have been inconsistent. Evidence of the association of parathyroidectomy (PTX) with these outcomes is also limited because of the lack of large well-controlled trials.

**Objective:**

To investigate whether untreated pHPT was associated with an increased risk of incident fractures and cardiovascular events (CVEs) and whether PTX was associated with a reduced risk of these outcomes.

**Design, Setting, and Participants:**

This cohort study included all patients who were diagnosed with pHPT at hospitals in Sweden between July 1, 2006, and December 31, 2017. Each patient was matched with 10 control individuals from the general population by sex, birth year, and county of residence. The patients were followed up until December 31, 2017. Data analyses were performed from October 2021 to April 2022.

**Main Outcomes and Measures:**

The primary outcomes were fractures, CVEs, and death. Cumulative incidence of events was estimated using the 1-minus Kaplan-Meier estimator of corresponding survival function. Cox proportional hazards regression models were used to calculate hazard ratios (HRs).

**Results:**

A total of 16 374 patients with pHPT were identified (mean [SD] age, 67.5 [12.9] years; 12 806 women [78.2%]), with 163 740 control individuals. The follow-up time was 42 310 person-years for the pHPT group and 803 522 person-years for the control group. Compared with the control group, the pHPT group had a higher risk of any fracture (unadjusted HR, 1.39; 95% CI, 1.31-1.48), hip fracture (unadjusted HR, 1.51; 95% CI, 1.35-1.70), CVEs (unadjusted HR, 1.45; 95% CI, 1.34-1.57), and death (unadjusted HR, 1.72; 95% CI, 1.65-1.80). In a time-dependent Poisson regression model, PTX was associated with a reduced risk of any fracture (HR, 0.83; 95% CI, 0.75-0.93), hip fracture (HR, 0.78; 95% CI, 0.61-0.98), CVEs (HR, 0.84; 95% CI, 0.73-0.97), and death (HR, 0.59; 95% CI, 0.53-0.65).

**Conclusions and Relevance:**

Results of this study suggest that pHPT is associated with increased risk of fractures, CVEs, and death, highlighting the importance of identifying patients with this condition to prevent serious unfavorable outcomes. The reduced risk of these outcomes associated with PTX suggests a clinical benefit of surgery.

## Key Points

**Question:**

Is the risk of fractures and cardiovascular events higher in patients with untreated primary hyperparathyroidism (pHPT) and lower in patients who undergo parathyroidectomy (PTX)?

**Findings:**

In this cohort study of 16 374 Swedish patients with pHPT, the risk of hip fracture was significantly increased by 51%, of any fracture by 39%, and of cardiovascular events by 45% in patients with pHPT compared with a matched control group. The risk of these outcomes was significantly reduced with PTX.

**Meaning:**

Findings from this study suggest an association between pHPT and increased risk of fractures, cardiovascular events, and death as well as an association between PTX and reduced risk of these outcomes.

## Teaser

This cohort study examines the association of fractures, cardiovascular events, and mortality with untreated primary hyperparathyroidism and with surgical treatment.

## Introduction

Primary hyperparathyroidism (pHPT) is a common endocrine disorder characterized by elevated serum calcium combined with normal or high blood levels of parathyroid hormone.^1^ The reported prevalence in the general population has varied between countries and over time. The prevalence of pHPT was 0.86% in the general population in a large US study^2^ and was 3.4% in a population-based screening program of Swedish postmenopausal women.^3^

In most high-income countries wherein biochemical screening of serum calcium is common, pHPT usually presents as an asymptomatic disorder of older adults.^4^ Disturbed calcium homeostasis can potentially have long-term adverse outcomes, including tissue damage in known target organs such as the kidneys, bone, and cardiovascular system.^5^ Observational studies of pHPT, especially severe cases, have reported a higher prevalence of osteoporosis, hypertension, diabetes, lipid abnormalities, endothelial dysfunction, arrhythmias, and left ventricular hypertrophy in patients with the disorder than in control individuals.^6,7^

Some population-based studies have observed a higher risk of cardiovascular events (CVEs) and death in patients with pHPT,^8,9,10^ a conclusion supported by a meta-analysis of prospective studies.^11^ International guidelines on pHPT management do not include cardiovascular disease among the criteria for parathyroidectomy (PTX)^12^ because of insufficient evidence regarding the association of PTX with cardiovascular risk.^13,14,15^

Increased bone loss and an elevated fracture risk at most skeletal sites, including the spine, wrist, ribs, and pelvis, are known concerns in pHPT.^16^ For hip fracture, the most severe fracture associated with substantially higher morbidity and mortality, evidence of increased risk is insufficient and inconsistent.^17,18,19,20^ The American Association of Endocrine Surgeons guidelines for management of pHPT recommend PTX in patients with symptomatic pHPT, hypercalcemia (>1 mg/dL above the normal range), kidney involvement, or osteoporosis or fragility fracture.^10^

Because pHPT is easily diagnosed and diagnosis may be important for identifying the future risk of severe and debilitating outcomes, including hip fractures, CVEs, and mortality, additional well-powered and representative population-based studies with incident outcome data are warranted. In this retrospective cohort study, we aimed to investigate whether untreated pHPT was associated with an increased risk of incident fractures (including hip fractures) and CVEs compared with the risk of the sex, birth year, and county–matched control group. We also sought to investigate whether PTX was associated with a reduced risk of these outcomes vs nonoperatively managed pHPT.

## Methods

### Study Design

The cohort study was approved by the regional ethical review board of Gothenburg, Sweden, which issued a waiver of the patient informed consent requirement because all of the data used were collected from registers without the investigators having direct contact with participants. We followed the Strengthening the Reporting of Observational Studies in Epidemiology (STROBE) reporting guideline.

All patients who were diagnosed with pHPT (*International Statistical Classification of Diseases and Related Health Problems, Tenth Revision [ICD-10]* code E210) at hospitals in Sweden between July 1, 2006, and December 31, 2017, were included. To study untreated patients with pHPT exclusively, we excluded patients who were previously treated with antiparathyroid agents (Anatomical Therapeutic Chemical code H05BX [eg, cinacalcet]) or PTX (Klassifikation av kirurgiska åtgärder codes BBA3-BBA5) before the pHPT diagnosis or study period. Each patient with pHPT was matched to 10 control individuals from the general population by sex, birth year, and county of residence using sampling with replacement to avoid bias.^21^ Control individuals were assigned the baseline diagnosis date of their matched patient; control individuals with a previous pHPT diagnosis who underwent PTX or who had treatment with antiparathyroid agents were excluded.

The risk of fractures, injurious falls, CVEs, kidney stones, and overall and cardiovascular deaths was analyzed. Follow-up time was censored at the end of study (December 31, 2017), emigration, or death (whichever occurred first) and also at the start of antiparathyroid agents or PTX and osteoporosis medication (for fracture outcomes). Control individuals were also censored at incident pHPT, PTX, or use of antiparathyroid agents. Associations between PTX and different outcomes were investigated separately in patients with pHPT, with PTX serving as a time-dependent variable.

### Data Sources

Swedish national registers were combined to serve as sources of clinical characteristics and outcomes of patients with pHPT. All Swedish residents are assigned a unique personal identification number at birth or at the time of immigration, enabling data linkage across the national registers. The Swedish Patient Register was used to retrieve data on pHPT diagnosis, comorbidities, CVEs, kidney stones, fractures, and injurious falls that were identified during inpatient and outpatient visits. Medication data were retrieved from the Swedish Prescribed Drug Register, and data on socioeconomic factors and deaths were obtained from Statistics Sweden. Race and ethnicity data were not available in the Swedish registers used.

### Variable Definitions

Data on fractures, cardiovascular outcomes, and comorbidities were collected from hospital visits and were defined using *ICD-10* codes (eTable 1 in the Supplement). All nonmalignant fracture diagnoses regardless of type of trauma were included, and the data were refined in several steps. Fracture diagnoses with a simultaneous code indicating a revisit (*ICD-10* codes Z09, Z47, and Z48) and hip fracture diagnoses without a simultaneous code for surgical procedure (Klassifikation av kirurgiska åtgärder codes NFB, NFC, or NFJ) were discarded. Furthermore, a washout period of 5 months was used; that is, if a fracture diagnosis referring to the same skeletal site was repeated within 5 months, the latter diagnosis was excluded, minimizing the role of revisits and maximizing the accuracy of true fractures.^22^ Incident hip fracture was defined as a fracture in the femoral head, neck, trochanter, or subtrochanteric region of the femur. Incident fracture of any kind included all fractures except of the head, fingers, and toes. Incident major osteoporotic fracture was defined as a fracture of the hip, vertebrae, pelvis, upper arm, or lower arm.

To avoid overlap with the fracture outcomes, we specified injurious fall as any event with an injury code (*ICD-10* codes S00-T14, with a 5-month washout period) and a fall code (*ICD-10* codes W00-W19) but without a fracture code. Incident CVE included admissions with myocardial infarction (*ICD-10* codes I21-I22), hemorrhagic stroke (*ICD-10* code I63), or ischemic stroke (*ICD-10* code I61), with a 7-year washout period for each diagnosis.^23^ For incident kidney stone (*ICD-10* codes N20-N22), a washout period of 1 year was used.^24^

A number of covariates representing previous illnesses and medications with presumed implications for a patient’s comorbidity and risk of the studied outcomes were selected (Table 1). Charlson Comorbidity Index was calculated to summarize and quantify comorbidity.^25^ This index ranged from 0 to 15, with the lowest score indicating very low 1-year mortality rates.   

**Table 1.** Baseline Characteristics

Variable^a^	Individuals, No. (%)		SMD
	Control group (n = 163 740)	pHPT group (n = 16 374)	
Age, mean (SD), y	67.5 (12.9)	67.5 (12.9)	0.000
Sex			
Female	128 060 (78.2)	12 806 (78.2)	0.000
Male	35 680 (21.8)	3568 (21.8)	0.000
Urban residency, ≥200 per km^2^	44 627 (27.3)	4774 (29.2)	0.042
Sickness benefits	9340 (5.7)	2209 (13.5)	0.267
Non-Nordic citizenship at birth	13 450 (8.2)	1442 (8.8)	0.021
Charlson Comorbidity Index			
Mean (SD)	0.61 (1.32)	0.93 (1.57)	0.218
0	118 665 (72.5)	9823 (60.0)	−0.266
1 or 2	34 032 (20.8)	4636 (28.3)	0.176
≥3	11 043 (6.7)	1915 (11.7)	0.172
Diagnoses^b^			
Osteoporosis	3775 (2.3)	1204 (7.4)	0.237
Previous alcohol-related disease	1740 (1.1)	188 (1.1)	0.008
Rheumatoid arthritis	2523 (1.5)	378 (2.3)	0.056
Previous fracture			
Any	17 405 (10.6)	2484 (15.2)	0.136
Recent (past year)	4672 (2.9)	994 (6.1)	0.156
Multiple (≥2 occasions)	3194 (2.0)	499 (3.0)	0.070
Previous injurious fall			
Any	10 267 (6.3)	1412 (8.6)	0.090
Recent (past year)	2722 (1.7)	477 (2.9)	0.084
Multiple (≥2 occasions)	1172 (0.7)	176 (1.1)	0.038
Dementia	2862 (1.7)	325 (2.0)	0.018
Ischemic heart disease	10 953 (6.7)	1646 (10.1)	0.122
Myocardial infarction	3025 (1.8)	455 (2.8)	0.062
Cerebrovascular disease			
Any	6459 (3.9)	927 (5.7)	0.080
Previous hemorrhagic stroke	388 (0.2)	64 (0.4)	0.028
Previous ischemic stroke	3091 (1.9)	457 (2.8)	0.060
CHD	5545 (3.4)	1036 (6.3)	0.137
Diabetes	10 563 (6.5)	1720 (10.5)	0.146
Kidney failure	1890 (1.2)	716 (4.4)	0.197
Previous kidney stone	1731 (1.1)	984 (6.0)	0.271
COPD	7010 (4.3)	1154 (7.0)	0.120
Hyperthyroidism	1207 (0.7)	369 (2.3)	0.125
Medications used in past year^c^			
Osteoporosis drugs	7758 (4.7)	1079 (6.6)	0.080
Calcium and vitamin D	10 288 (6.3)	662 (4.0)	−0.101
Prednisolone	11 286 (6.9)	1637 (10.0)	0.112
Opioids	10 896 (6.7)	1769 (10.8)	0.147
Antiepileptics	3859 (2.4)	576 (3.5)	0.069
Antiparkinson drugs	2234 (1.4)	273 (1.7)	0.025
Antidepressants	18 818 (11.5)	2467 (15.1)	0.105
Antidementia drugs	2022 (1.2)	147 (0.9)	−0.033
Thiazid diuretics	7816 (4.8)	873 (5.3)	0.025
β-blockers	35 053 (21.4)	4536 (27.7)	0.147
Calcium antagonist	20 999 (12.8)	2960 (18.1)	0.146
RAS inhibitors	37 649 (23.0)	5319 (32.5)	0.213

Abbreviations: CHD, congestive heart disease; COPD, chronic obstructive pulmonary disease; pHPT, primary hyperparathyroidism; RAS, renin-angiotensin system; SMD, standardized mean difference.

^a^ Detailed definitions of variables are presented in eTable 1 in the Supplement.

^b^ A 5-year historical window was used for diagnoses, if not otherwise stated.

^c^ Medications used in the past year were defined by a prescription during the past year, were repeated (≥2 prescriptions), and were ongoing (most recent prescription collected <120 days before baseline).

### Statistical Analysis

To assess the differences in baseline characteristics, standardized mean differences were calculated. Incident rates per 1000 person-years were calculated to enable comparison of periods with different follow-up lengths and were presented with exact Poisson 95% CIs. The cumulative incidence of events was estimated using the 1-minus Kaplan-Meier estimator of the corresponding survival function and then presented graphically with 95% CIs. Cox proportional hazards regression models were used to calculate hazard ratios (HRs). Cox assumption of proportional hazards was tested using graphical methods. Cox proportional hazards regression models were adjusted for age, sex, Charlson Comorbidity Index, and prevalence of investigated outcome (previous fracture, injurious fall, CVE, or kidney stone).

A sensitivity analysis was performed, with the follow-up time censored after 1 year, reducing the difference in follow-up time between the pHTP group and the control group. Interaction and subgroup analyses stratified by sex and Charlson Comorbidity Index were performed. The potential association between PTX and outcomes was investigated using an extension of a Poisson regression model,^26,27^ which was adjusted for current time since diagnosis, current age, sex, calendar year, Charlson Comorbidity Index, and baseline prevalence of the investigated outcome. An alternative sensitivity analysis was conducted to compare the incidence of outcomes before and after surgery in patients who underwent PTX. To assess the potential implications of death as a competing risk, we estimated the cumulative incidence function (or subdistribution function) of fracture or injurious fall, with death as a competing risk, using the Aalen-Johansen estimator. The subdistribution hazard for fracture was compared between the pHPT group and control group (matched 1:1) using the Fine and Gray proportional hazards model, with death as the competing risk.^28^

Statistical analyses were performed from October 2021 to April 2022, using SPSS, version 26 (IBM SPSS), and RStudio, version 1.4.1106 (R Foundation for Statistical Computing). Two-sided *P* < .05 (*P* = .10 for interactions) was considered to be significant.

## Results

In total, 16 374 hospital patients with pHPT were identified, mostly during outpatient visits. These patients included 12 806 women (78.2%) and 3568 men (21.8%) with a mean (SD) age of 67.5 (12.9) years. Each patient was matched to 10 control individuals by sex, birth year, and county of residence (n = 163 740). Baseline characteristics of the pHPT and control groups are presented in Table 1.

Patients in the pHPT group had more comorbidities than the control group (Charlson Comorbidity Index ≥3: 11.7% vs 6.7%). Similarly, the proportion with registered sickness benefits was higher among the pHPT group than among the control group (13.5% vs 5.7%). Both previous fracture (15.2% vs 10.6%) and injurious fall (8.6% vs 6.3%) were more common among patients with pHPT than among control individuals. Previous diagnosis of osteoporosis was documented in 7.4% of patients with pHPT compared with 2.3% of the control individuals. A smaller percentage of patients in the pHPT group vs control group had received a prescription for calcium and vitamin D supplements (4.0% vs 6.3%), but use of other medications for a variety of conditions was consistently higher in the pHPT group than in the control group. A greater proportion of patients with pHPT than control individuals had experienced a previous myocardial infarction (2.8% vs 1.8%) and were more likely to have been diagnosed with ischemic heart disease (10.1% vs 6.7%) and cerebrovascular disease (5.7% vs 3.9%). The prevalence of kidney stones (6.0% vs 1.1%) and kidney failure (4.4% vs 1.2%) was higher in the pHPT group vs the control group (Table 1).

The total follow-up time for the study was 845 832 person-years, with 42 310 person-years for the pHPT group and 803 522 person-years for the control group. The median (IQR) follow-up time was 1.15 (0.40-4.06) years for the pHPT group and 4.62 (2.08-7.51) years for the control group. More than half of patients with pHPT were censored before the end of the study or death from either parathyroidectomy (n = 6934 [42.3%]; median [IQR] time, 0.50 [0.25-0.94] years) or cinacalcet treatment (n = 943 [5.8%]; median [IQR] time, 0.71 [0.17-2.74] years) or, for fracture outcomes, from osteoporosis treatment (n = 3283 [20.1%]; median [IQR] time, 0.38 [0.11-2.01] years).

During follow-up, the incidence rates per 1000 person-years for any fracture (35.52 [95% CI, 33.50-37.64] vs 25.36 [95% CI, 24.98-25.74]), major osteoporotic fracture (23.46 [95% CI, 21.85-25.17] vs 16.35 [95% CI, 16.05-16.65]), hip fracture (9.30 [95% CI, 8.31-10.38] vs 6.26 [95% CI, 6.08-6.45]), injurious fall (28.59 [95% CI, 26.95-30.31] vs 18.97 [95% CI, 18.66-19.28]), any CVE (17.22 [95% CI, 15.97-18.54] vs 11.98 [95% CI, 11.74-12.22]), kidney stone (9.27 [95% CI, 8.37-10.25] vs 2.47 [95% CI, 2.36-2.58]), and death (51.83 [95% CI, 49.68-54.05] vs 30.95 [95% CI, 30.57-31.34]) were significantly higher in patients with pHPT than in control individuals (Table 2). The risk was also significantly higher for all observed outcomes, as demonstrated by unadjusted HRs for the pHPT group vs the control group (Table 2; Figure). Patients with pHPT had significantly higher risk of any fracture (unadjusted HR, 1.39; 95% CI, 1.31-1.48), major osteoporotic fracture (unadjusted HR, 1.43; 95% CI, 1.33-1.54), hip fracture (unadjusted HR, 1.51; 95% CI, 1.35-1.70), and injurious fall (unadjusted HR, 1.51; 95% CI, 1.42-1.60) compared with control individuals. The risk of fractures was significantly increased at different peripheral sites (eTable 2 in the Supplement): wrist (unadjusted HR, 1.34; 95% CI, 1.18-1.52), upper arm (unadjusted HR, 1.46; 95% CI, 1.25-1.71), and lower leg (unadjusted HR, 1.31; 95% CI, 1.12-1.54). For any CVE, patients with pHPT had an overall increased risk (unadjusted HR, 1.45; 95% CI, 1.34-1.57), as well as increased risk for acute myocardial infarction (unadjusted HR, 1.39; 95% CI, 1.24-1.56) and for ischemic stroke (unadjusted HR, 1.51; 95% CI, 1.36-1.68), whereas there was no significantly increased risk for hemorrhagic stroke (unadjusted HR, 1.09; 95% CI, 0.82-1.45) (eTable 3 in the Supplement). The risk of overall deaths (unadjusted HR, 1.72; 95% CI, 1.65-1.80) and cardiovascular-related deaths was higher among the pHPT group than among the control group (eTable 4 in the Supplement). The risk of kidney stones during follow-up was almost 4 times higher in patients with pHPT than in control individuals (unadjusted HR, 3.65; 95% CI, 3.27-4.08). All associations remained significant, although attenuated after adjustment for age, sex, Charlson Comorbidity Index, and prevalence of investigated outcomes.

**Table 2.** Clinical Outcomes

Variable	Control group (n = 163 740)	pHPT group (n = 16 374)^a^	*P* value
Time at risk, median (IQR), y	4.62 (2.08-7.51)	1.15 (0.40-4.06)	NA
Any fracture			
Events, No. (%)	17 326 (10.6)	1150 (7.0)	NA
Per 1000 person-years (95% CI)	25.36 (24.98-25.74)	35.52 (33.50-37.64)	
Cox proportional hazards regression model, HR (95% CI)^b^			
Unadjusted	1 [Reference]	1.39 (1.31-1.48)	<.001
Adjusted	1 [Reference]	1.22 (1.15-1.30)	<.001
Major osteoporotic fracture			
Events, No. (%)	11 472 (7.0)	780 (4.8)	NA
Per 1000 person-years (95% CI)	16.35 (16.05-16.65)	23.46 (21.85-25.17)	
Cox proportional hazards regression model, HR (95% CI)^b^			
Unadjusted	1 [Reference]	1.43 (1.33-1.54)	<.001
Adjusted	1 [Reference]	1.23 (1.15-1.33)	<.001
Hip fracture			
Events, No. (%)	4519 (2.8)	319 (1.9)	NA
Per 1000 person-years (95% CI)	6.26 (6.08-6.45)	9.30 (8.31-10.38)	
Cox proportional hazards regression model, HR (95% CI)^b^			
Unadjusted	1 [Reference]	1.51 (1.35-1.70)	<.001
Adjusted	1 [Reference]	1.20 (1.07-1.35)	.002
Injurious fall			
Events, No. (%)	14 436 (8.8)	1130 (6.9)	NA
Per 1000 person-years (95% CI)	18.97 (18.66-19.28)	28.59 (26.95-30.31)	
Cox proportional hazards regression model, HR (95% CI)^b^			
Unadjusted	1 [Reference]	1.51 (1.42-1.60)	<.001
Adjusted	1 [Reference]	1.30 (1.22-1.38)	<.001
Any CVE			
Events, No. (%)	9350 (5.7)	703 (4.3)	NA
Per 1000 person-years (95% CI)	11.98 (11.74-12.22)	17.22 (15.97-18.54)	
Cox proportional hazards regression model, HR (95% CI)^b^			
Unadjusted	1 [Reference]	1.45 (1.34-1.57)	<.001
Adjusted	1 [Reference]	1.22 (1.13-1.32)	<.001
Kidney stone			
Events, No. (%)	1967 (1.2)	384 (2.3)	NA
Per 1000 person-years (95% CI)	2.47 (2.36-2.58)	9.27 (8.37-10.25)	
Cox proportional hazards regression model, HR (95% CI)^b^			
Unadjusted	1 [Reference]	3.65 (3.27-4.08)	<.001
Adjusted	1 [Reference]	2.60 (2.32-2.91)	<.001
Death			
Events, No. (%)	24 869 (15.2)	2193 (13.4)	NA
Per 1000 person-years (95% CI)	30.95 (30.57-31.34)	51.83 (49.68-54.05)	
Cox proportional hazards regression model, HR (95% CI)^b^			
Unadjusted	1 [Reference]	1.72 (1.65-1.80)	<.001
Adjusted	1 [Reference]	1.27 (1.22-1.33)	<.001
Cardiovascular-related deaths			
Events, No. (%)	8106 (5.0)	722 (4.4)	NA
Per 1000 person-years (95% CI)	10.09 (9.87-10.31)	17.06 (15.84-18.36)	
Cox proportional hazards regression model, HR (95% CI)^b^			
Unadjusted	1 [Reference]	1.73 (1.60-1.86)	<.001
Adjusted	1 [Reference]	1.24 (1.15-1.34)	<.001

Abbreviations: CVE, cardiovascular event; HR, hazard ratio; NA, not applicable; pHPT, primary hyperparathyroidism.

^a^ All patients in the pHPT group were included and censored before the end of the study period or death from either parathyroidectomy or cinacalcet treatment or, for the fracture outcomes, from osteoporosis treatment.

^b^ Cox proportional hazards regression models were adjusted for age, sex, Charlson Comorbidity Index, and the prevalence of the investigated outcome (previous fracture, injurious fall, CVE, or kidney stone).

**Figure.**
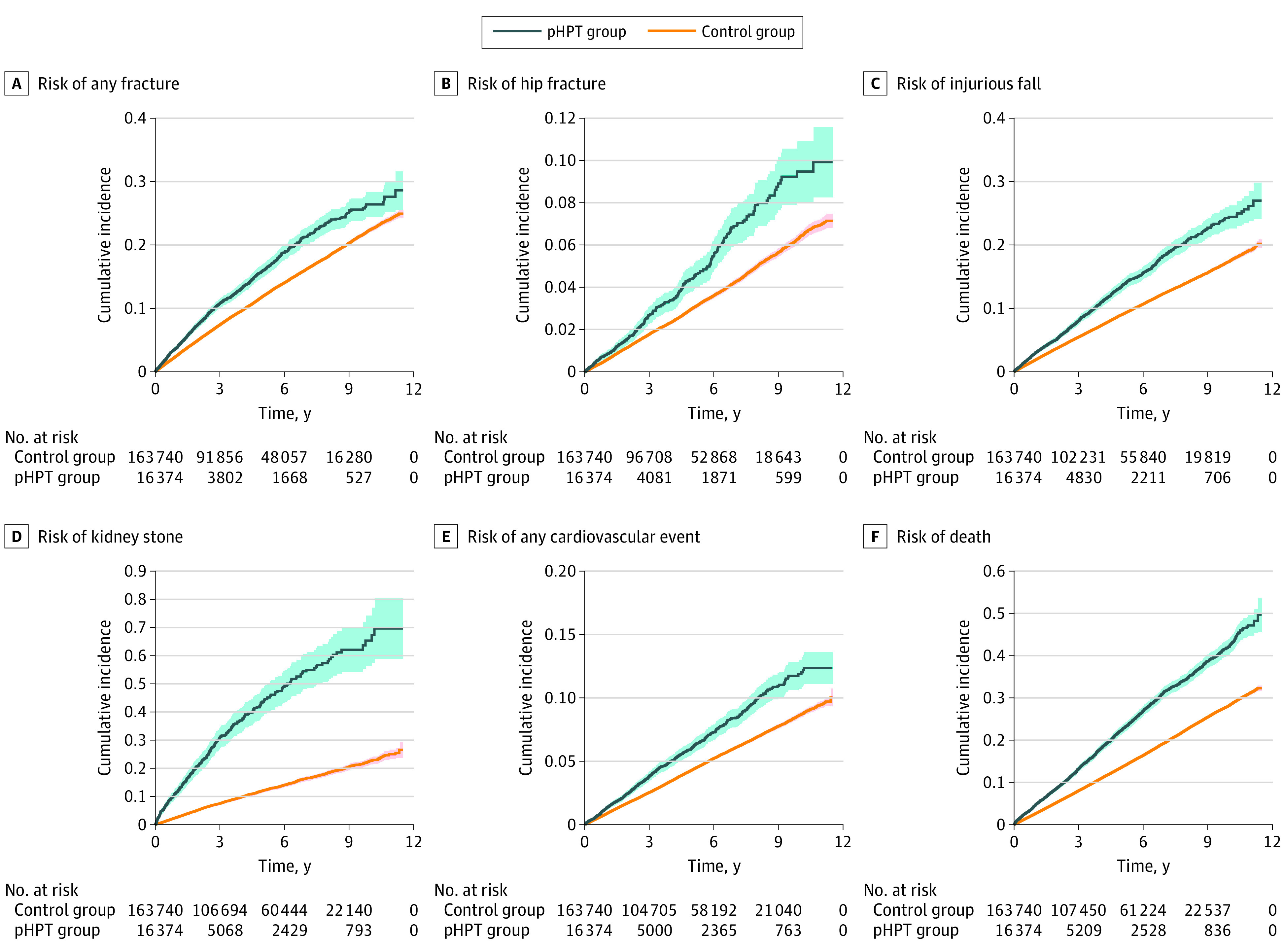
Cumulative Incidences for Patients With Primary Hyperparathyroidism (pHPT) and Control Individuals The cumulative incidence of events for untreated pHPT and control groups was estimated using 1-minus Kaplan-Meier estimate of the corresponding survival function and presented with 95% CIs (indicated by shaded areas).

There was no interaction between pHPT diagnosis and sex for any outcome, and the HRs for all reported outcomes in unadjusted analyses were significant in both men and women with pHPT compared with control individuals (eTable 5 in the Supplement). There was no interaction between pHPT diagnosis and Charlson Comorbidity Index for any fracture or CVE.

To account for the difference in follow-up time between groups, we performed a sensitivity analysis in which the Cox proportional hazards regression models were truncated after 1 year. The increased risk of all outcomes in the pHPT group was significant and similar to that found in the main analysis (eTable 6 in the Supplement). For example, the risk of hip fracture was 51% higher both in the analysis with truncated follow-up (HR, 1.51; 95% CI, 1.21-1.87) and in the analysis using the entire follow-up (HR, 1.51; 95% CI, 1.35-1.70).

Visualization of the cumulative incidence functions of each outcome, with death as a competing risk, revealed similar incidence differences in the studied associations (eFigure in the Supplement). Fine and Gray analyses with death as a competing risk demonstrated that the associations between pHPT and all outcomes remained (eTable 7 in the Supplement). For example, the risk of hip fracture was increased after adjustment for competing risk (sub-HR, 1.37; 95% CI, 1.19-1.58).

Of the 16 374 patients with pHPT, 6934 (42.3%) underwent PTX a median (IQR) of 0.50 (0.25-0.94) years after diagnosis. These patients were considerably younger, had substantially fewer comorbidities, and had lower prevalence of fracture and CVE compared with patients who received conservative treatment (Table 3).

**Table 3.** Baseline Characteristics per Conservative Treatment or Future Parathyroidectomy

Variable^a^	Individuals, No. (%)		SMD
	Conservative treatment	Parathyroidectomy	
No. of patients	9440	6934	NA
Age, mean (SD), y	71.1 (12.5)	62.6 (11.6)	−0.710
Female sex	7418 (78.6)	5388 (77.7)	−0.021
Male sex	2022 (21.4)	1546 (22.3)	0.021
Urban residency, ≥200 per km^2^	2768 (29.3)	2006 (28.9)	−0.009
Sickness benefits	815 (8.6)	1394 (20.1)	0.331
Non-Nordic citizenship at birth	829 (8.8)	613 (8.8)	0.002
Charlson Comorbidity Index			
Mean (SD)	1.18 (1.76)	0.58 (1.19)	−0.403
0	4869 (51.6)	4954 (71.4)	0.417
1 or 2	3060 (32.4)	1576 (22.7)	−0.218
≥3	1511 (16.0)	404 (5.8)	−0.331
Diagnoses^b^			
Osteoporosis	836 (8.9)	368 (5.3)	−0.139
Previous alcohol-related disease	111 (1.2)	77 (1.1)	−0.006
Rheumatoid arthritis	251 (2.7)	127 (1.8)	−0.056
Previous fracture			
Any	1641 (17.4)	843 (12.2)	−0.148
Recent (past year)	688 (7.3)	306 (4.4)	−0.123
Multiple (≥2 occasions)	354 (3.8)	145 (2.1)	−0.099
Previous injurious fall			
Any	920 (9.7)	492 (7.1)	−0.096
Recent (past year)	352 (3.7)	125 (1.8)	−0.118
Multiple (≥2 occasions)	128 (1.4)	48 (0.7)	−0.066
Dementia	306 (3.2)	19 (0.3)	−0.227
Ischemic heart disease	1225 (13.0)	421 (6.1)	−0.237
Myocardial infarction	359 (3.8)	96 (1.4)	−0.153
Cerebrovascular disease			
Any	728 (7.7)	199 (2.9)	−0.218
Previous hemorrhagic stroke	52 (0.6)	12 (0.2)	−0.063
Previous ischemic stroke	356 (3.8)	101 (1.5)	−0.145
CHD	891 (9.4)	145 (2.1)	−0.319
Diabetes	1228 (13.0)	492 (7.1)	−0.198
Kidney failure	552 (5.8)	164 (2.4)	−0.176
Previous kidney stone	437 (4.6)	547 (7.9)	0.135
COPD	844 (8.9)	310 (4.5)	−0.179
Hyperthyroidism	254 (2.7)	115 (1.7)	−0.071
Medications used in past year^c^			
Osteoporosis drugs	711 (7.5)	368 (5.3)	−0.091
Calcium and vitamin D	502 (5.3)	160 (2.3)	−0.158
Prednisolone	1124 (11.9)	513 (7.4)	−0.153
Opioids	1184 (12.5)	585 (8.4)	−0.134
Antiepileptics	389 (4.1)	187 (2.7)	−0.079
Antiparkinson drugs	207 (2.2)	66 (1.0)	−0.100
Antidepressants	1568 (16.6)	899 (13.0)	−0.103
Antidementia drugs	128 (1.4)	19 (0.3)	−0.121
Thiazide diuretics	565 (6.0)	308 (4.4)	−0.069
β-blockers	3072 (32.5)	1464 (21.1)	−0.260
Calcium antagonist	1906 (20.2)	1054 (15.2)	−0.131
RAS inhibitors	3465 (36.7)	1854 (26.7)	−0.215

Abbreviations: CHD, congestive heart disease; COPD, chronic obstructive pulmonary disease; NA, not applicable; RAS, renin-angiotensin system; SMD, standardized mean difference.

^a^ Detailed definitions of variables are provided in eTable 1 in the Supplement.

^b^ A 5-year historical window was used for diagnoses, if not otherwise stated.

^c^ Medications used in the past year were defined by a prescription during the past year, were repeated (≥2 prescriptions), and were ongoing (most recent prescription collected <120 days before baseline).

When we studied the pHPT group separately using an extension of the Poisson regression model, with incident PTX as a time-dependent variable, we found an association between PTX and reduced risk of hip fracture (HR, 0.78; 95% CI, 0.61-0.98), any fracture (HR, 0.83; 95% CI, 0.75-0.93), injurious fall (HR, 0.83; 95% CI, 0.74-0.92), CVE (HR, 0.84; 95% CI, 0.73-0.97), and death (HR, 0.59; 95% CI, 0.53-0.65) after adjustment for time since diagnosis, current age, sex, and calendar year. These associations remained similar after further adjustment for prevalent disease and Charlson Comorbidity Index (Table 4).

**Table 4.** Association Between Parathyroidectomy and Outcomes^a^

	HR (95% CI)	*P* value
Any fracture		
Adjusted for time since diagnosis, current age, sex, and calendar year	0.83 (0.75-0.93)	.001
Further adjusted for Charlson Comorbidity Index and previous fracture	0.84 (0.76-0.94)	.003
Major osteoporotic fracture		
Adjusted for time since diagnosis, current age, sex, and calendar year	0.82 (0.71-0.94)	.004
Further adjusted for Charlson Comorbidity Index and previous fracture	0.83 (0.72-0.95)	.007
Hip fracture		
Adjusted for time since diagnosis, current age, sex, and calendar year	0.78 (0.61-0.98)	.04
Further adjusted for Charlson Comorbidity Index and previous fracture	0.80 (0.63-1.02)	.07
Injurious fall		
Adjusted for time since diagnosis, current age, sex, and calendar year	0.83 (0.74-0.92)	<.001
Further adjusted for Charlson Comorbidity Index and previous injurious fall	0.85 (0.76-0.95)	.003
Any CVE		
Adjusted for time since diagnosis, current age, sex, and calendar year	0.84 (0.73-0.97)	.02
Further adjusted for Charlson Comorbidity Index and previous CVE	0.87 (0.75-1.00)	.05
Acute myocardial infarction		
Adjusted for time since diagnosis, current age, sex, and calendar year	0.84 (0.68-1.04)	.10
Further adjusted for Charlson Comorbidity Index and previous CVE	0.87 (0.70-1.08)	.20
Ischemic stroke		
Adjusted for time since diagnosis, current age, sex, and calendar year	0.90 (0.74-1.09)	.28
Further adjusted for Charlson Comorbidity Index and previous CVE	0.92 (0.76-1.12)	.30
Kidney stone		
Adjusted for time since diagnosis, current age, sex, and calendar year	0.89 (0.76-1.06)	.19
Further adjusted for Charlson Comorbidity Index and previous kidney stone	0.77 (0.65-0.91)	.003
Death overall		
Adjusted for time since diagnosis, current age, sex, and calendar year	0.59 (0.53-0.65)	<.001
Further adjusted for Charlson Comorbidity Index	0.64 (0.58-0.71)	<.001
Cardiovascular-related death		
Adjusted for time since diagnosis, current age, sex, and calendar year	0.60 (0.50-0.71)	<.001
Further adjusted for Charlson Comorbidity Index and previous CVE	0.71 (0.59-0.85)	<.001

Abbreviations: CVE, cardiovascular event; HR, hazard ratio.

^a^ Parathyroidectomy was used as a time-dependent variable in the Poisson regression model, with adjustments for time since diagnosis, current age, sex, and calendar year in the primary models as well as outcome-specific additional adjustments as indicated.

When we investigated the outcomes before and after surgery of the 6934 patients who underwent PTX, using a Cox proportional hazards regression model adjusted for age and sex, we found that the risk of any fracture (HR, 0.77; 95% CI, 0.63-0.95) and kidney stone (HR, 0.61; 95% CI, 0.48-0.78) was lower after vs before PTX, whereas no risk difference was seen for injurious fall (HR, 1.02; 95% CI, 0.80-1.31) (eTable 8 in the Supplement).

## Discussion

Patients with pHPT had a significantly higher risk of hip fracture, CVE, and all-cause mortality vs their control counterparts. In patients with pHPT, PTX was associated with reduced risk of all of these outcomes.

Although pHPT is a relatively common disorder,^1^ especially among postmenopausal women, and its clinical consequences for affected organ systems have long been known, there is still controversy regarding the clinical importance of these outcomes. Most studies of fracture risk in pHPT are relatively small, and a recent meta-analysis of all potential publications between 1966 and 2019 revealed only 12 studies involving 5233 patients with pHPT and 13 154 control individuals.^20^ The meta-analysis reported an increased risk of any fracture, but the analysis of hip fracture risk was limited to 3 studies that included relatively young patients with pHPT; thus, few cases of hip fractures emerged during the follow-up period. This limitation likely affected the observed lack of association between pHPT and risk of hip fracture.^18,19,29^

To our knowledge, in terms of the number of patients with pHPT and observed outcomes, the present cohort study is the largest analysis performed thus far. The 51% increased risk of hip fracture in patients with pHPT (unadjusted HR, 1.51) compared with control individuals was based on 319 and 4519 events, respectively. Hip fracture is the most serious osteoporosis-related fracture and is associated with substantially higher morbidity, mortality, and functional decline,^30^ emphasizing the importance of identifying patients at high risk and including pHPT in the risk assessment.

Previous studies found impaired bone properties in both trabecular and cortical bone^31^ in patients with pHPT. This finding was supported by a recent meta-analysis that identified an increased risk of any fracture, vertebral fracture, and forearm fracture in these patients.^20^ Results of the present study, which used a substantially larger data set, are in agreement with these previous analyses. We found an increased risk of any fracture, major osteoporotic fracture, and peripheral fracture in patients with pHPT.

Randomized clinical trials in patients with mild pHPT found that PTX improved bone mineral density and reduced serum calcium, but its role in the risk of fracture could not be ascertained owing to the small number of patients included in these trials.^32^ Observational studies found that the risk of fracture appeared to be lower after rather than before surgery,^17^ but this finding was confounded by the observation that those who underwent PTX had lower risk than those who were treated conservatively.^33^ In the time-dependent analysis in the present study, PTX was associated with a decrease in fracture risk. This finding was supported by the lower fracture rate and fracture risk seen after rather than before surgery, further supporting that PTX was associated with lower fracture risk. This finding also agrees with results from a recent observational study of 183 433 Medicare beneficiaries in the US.^34^ A lower fracture rate was observed in patients with pHPT (87.3% women) who underwent PTX compared with those who had conservative treatment.^34^ Because control individuals without pHPT were not included, the risk of fracture associated with pHPT was not analyzed.^34^

Previous studies of surgical cases and untreated patients with pHPT found an increased risk of myocardial events and cardiovascular mortality^8,35,36,37^ in both of these groups. This finding was in contrast to the lack of elevated mortality risk reported by a US study of patients with pHPT in the community.^9^ In a Danish cohort of 674 patients with pHPT who underwent PTX and an equal number of sex- and age-matched control individuals, the risk of myocardial infarction, stroke, congestive heart failure, and hypertension was higher before surgery; more than a year after surgery, this increased risk was no longer seen for myocardial infarction and stroke but remained for the other outcomes. However, the Danish study was limited by few events for several outcomes.^38^ Because of the conflicting results, especially regarding mild pHPT, and the absence of large randomized clinical trials, there is insufficient evidence to recommend PTX based on risk of cardiovascular disease.^10^ In the present study, the risk of myocardial infarction, stroke (both ischemic and hemorrhagic), and death was higher in untreated patients with pHPT and the risk was inversely associated with PTX. These findings support the inclusion of cardiovascular risk in the indications for surgery, but large randomized clinical trials are needed to identify any benefits of PTX.

With advancing age, the risks of functional decline, reduced muscle performance, and falls increase.^39^ Falls are the leading cause of injury-related morbidity and mortality in older men and women in the US.^40^ Nearly 29% of the population 65 years or older reported falling in the past year.^40^ The risk of falls or injurious falls in patients with pHPT has not been investigated. In this study, this risk was found to be inversely associated with PTX, indicating that falls may be prevented by surgery in some patients. We did not confirm this finding by analyzing the risk before and after PTX, but survival bias could have affected this analysis.

### Strengths and Limitations

This study has several strengths. To our knowledge, it is the largest study yet to investigate multiple outcomes in all untreated patients with pHPT identified in hospitals in Sweden as well as matched control individuals. A time-dependent model, which accounted in part for bias, was used to study the association between PTX and outcomes.

This study also has several limitations. First, the observational design could not establish causality, although the time-dependent analysis suggested an association between PTX and outcomes. Second, data on pHPT-related symptoms, serum calcium, or parathyroid hormone were not available, preventing the assessment of pHPT severity. Third, patients were included on the basis of hospital diagnoses; thus patients who were diagnosed in a primary care setting were not included. Fourth, the use of control individuals from the general population with less comorbidity than patients who were diagnosed with pHPT at hospitals could introduce a bias. However, the absence of a significant interaction between pHPT and Charlson Comorbidity Index for any fracture or CVE indicates an increased risk, regardless of other comorbidities.

## Conclusions

The increased risk of fractures, CVEs, and death observed in patients with pHPT suggests that it is important to identify patients with this condition to prevent serious outcomes. The reduced risk of these outcomes associated with PTX suggests a clinical benefit of surgery.

## Notes

Supplement. **eTable 1.** Definitions **eTable 2.** Other Fracture Outcomes **eTable 3.** Cardiovascular Outcomes **eTable 4.** Overall Death and Cardiovascular Deaths **eTable 5.** Clinical Outcomes per Sex **eTable 6.** Clinical Outcomes Within One Year **eFigure.** Cumulative Incidence Function in PHPT Patients vs. Controls **eTable 7.** Subhazard Ratios for PHPT Patients vs Population Controls, With Consideration of Competing Risk of Death **eTable 8.** Outcomes for PTX Patients After Surgery Using the Same PTX Patients Before Surgery as Controls

## References

[1] Bilezikian JP. Primary hyperparathyroidism. J Clin Endocrinol Metab. 2018;103(11):3993-4004. doi:10.1210/jc.2018-01225 30060226 PMC6182311

[2] Press DM, Siperstein AE, Berber E, . The prevalence of undiagnosed and unrecognized primary hyperparathyroidism: a population-based analysis from the electronic medical record. Surgery. 2013;154(6):1232-1237. doi:10.1016/j.surg.2013.06.051 24383100

[3] Lundgren E, Hagström EG, Lundin J, . Primary hyperparathyroidism revisited in menopausal women with serum calcium in the upper normal range at population-based screening 8 years ago. World J Surg. 2002;26(8):931-936. doi:10.1007/s00268-002-6621-0 12045863

[4] Silverberg SJ, Walker MD, Bilezikian JP. Asymptomatic primary hyperparathyroidism. J Clin Densitom. 2013;16(1):14-21. doi:10.1016/j.jocd.2012.11.005 23374736 PMC3987990

[5] Zhu CY, Sturgeon C, Yeh MW. Diagnosis and management of primary hyperparathyroidism. JAMA. 2020;323(12):1186-1187. doi:10.1001/jama.2020.0538 32031566

[6] Pepe J, Cipriani C, Sonato C, Raimo O, Biamonte F, Minisola S. Cardiovascular manifestations of primary hyperparathyroidism: a narrative review. Eur J Endocrinol. 2017;177(6):R297-R308. doi:10.1530/EJE-17-0485 28864535

[7] Assadipour Y, Zhou H, Kuo EJ, Haigh PI, Adams AL, Yeh MW. End-organ effects of primary hyperparathyroidism: a population-based study. Surgery. 2019;165(1):99-104. doi:10.1016/j.surg.2018.04.088 30420089

[8] Yu N, Donnan PT, Flynn RW, ; The Parathyroid Epidemiology and Audit Research Study (PEARS). Increased mortality and morbidity in mild primary hyperparathyroid patients. Clin Endocrinol (Oxf). 2010;73(1):30-34. doi:10.1111/j.1365-2265.2009.03766.x 20039887

[9] Wermers RA, Khosla S, Atkinson EJ, . Survival after the diagnosis of hyperparathyroidism: a population-based study. Am J Med. 1998;104(2):115-122. doi:10.1016/S0002-9343(97)00270-2 9528728

[10] Wilhelm SM, Wang TS, Ruan DT, . The American Association of Endocrine Surgeons guidelines for definitive management of primary hyperparathyroidism. JAMA Surg. 2016;151(10):959-968. doi:10.1001/jamasurg.2016.2310 27532368

[11] van Ballegooijen AJ, Reinders I, Visser M, Brouwer IA. Parathyroid hormone and cardiovascular disease events: a systematic review and meta-analysis of prospective studies. Am Heart J. 2013;165(5):655-664, 664.e1-664.e5. doi:10.1016/j.ahj.2013.02.014 23622902

[12] Khan AA, Hanley DA, Rizzoli R, . Primary hyperparathyroidism: review and recommendations on evaluation, diagnosis, and management—a Canadian and international consensus. Osteoporos Int. 2017;28(1):1-19. doi:10.1007/s00198-016-3716-2 27613721 PMC5206263

[13] Bollerslev J, Rosen T, Mollerup CL, ; SIPH Study Group. Effect of surgery on cardiovascular risk factors in mild primary hyperparathyroidism. J Clin Endocrinol Metab. 2009;94(7):2255-2261. doi:10.1210/jc.2008-2742 19351725

[14] Walker MD, Rundek T, Homma S, . Effect of parathyroidectomy on subclinical cardiovascular disease in mild primary hyperparathyroidism. Eur J Endocrinol. 2012;167(2):277-285. doi:10.1530/EJE-12-0124 22660025 PMC3668344

[15] Persson A, Bollerslev J, Rosen T, ; SIPH Study Group. Effect of surgery on cardiac structure and function in mild primary hyperparathyroidism. Clin Endocrinol (Oxf). 2011;74(2):174-180. doi:10.1111/j.1365-2265.2010.03909.x 21044114

[16] Lewiecki EM, Miller PD. Skeletal effects of primary hyperparathyroidism: bone mineral density and fracture risk. J Clin Densitom. 2013;16(1):28-32. doi:10.1016/j.jocd.2012.11.013 23374738

[17] Vestergaard P, Mollerup CL, Frøkjaer VG, Christiansen P, Blichert-Toft M, Mosekilde L. Cohort study of risk of fracture before and after surgery for primary hyperparathyroidism. BMJ. 2000;321(7261):598-602. doi:10.1136/bmj.321.7261.598 10977834 PMC27473

[18] Larsson K, Ljunghall S, Krusemo UB, Naessén T, Lindh E, Persson I. The risk of hip fractures in patients with primary hyperparathyroidism: a population-based cohort study with a follow-up of 19 years. J Intern Med. 1993;234(6):585-593. doi:10.1111/j.1365-2796.1993.tb01017.x 8258750

[19] Khosla S, Melton LJ III, Wermers RA, Crowson CS, O’Fallon WM, Riggs Bl. Primary hyperparathyroidism and the risk of fracture: a population-based study. J Bone Miner Res. 1999;14(10):1700-1707. doi:10.1359/jbmr.1999.14.10.1700 10491217

[20] Ejlsmark-Svensson H, Rolighed L, Harsløf T, Rejnmark L. Risk of fractures in primary hyperparathyroidism: a systematic review and meta-analysis. Osteoporos Int. 2021;32(6):1053-1060. doi:10.1007/s00198-021-05822-9 33527175

[21] Heide-Jørgensen U, Adelborg K, Kahlert J, Sørensen HT, Pedersen L. Sampling strategies for selecting general population comparison cohorts. Clin Epidemiol. 2018;10:1325-1337. doi:10.2147/CLEP.S164456 30310326 PMC6165733

[22] Axelsson KF, Jacobsson R, Lund D, Lorentzon M. Effectiveness of a minimal resource fracture liaison service. Osteoporos Int. 2016;27(11):3165-3175. doi:10.1007/s00198-016-3643-2 27230521 PMC5059408

[23] Socialstyrelsen. Statistik om hjärtinfarkter 2019. 2020. Accessed December 8, 2021. https://sdb.socialstyrelsen.se/if_hji/val.aspx

[24] Alexander RT, Hemmelgarn BR, Wiebe N, ; Alberta Kidney Disease Network. Kidney stones and kidney function loss: a cohort study. BMJ. 2012;345:e5287. doi:10.1136/bmj.e5287 22936784 PMC3431443

[25] Charlson ME, Pompei P, Ales KL, MacKenzie CR. A new method of classifying prognostic comorbidity in longitudinal studies: development and validation. J Chronic Dis. 1987;40(5):373-383. doi:10.1016/0021-9681(87)90171-8 3558716

[26] Albertsson-Wikland K, Mårtensson A, Sävendahl L, . Mortality is not increased in recombinant human growth hormone-treated patients when adjusting for birth characteristics. J Clin Endocrinol Metab. 2016;101(5):2149-2159. doi:10.1210/jc.2015-3951 26918292

[27] Breslow NE, Day NE. Statistical methods in cancer research: volume II—the design and analysis of cohort studies. IARC Sci Publ. 1987;(82):1-406. 3329634

[28] Fine JP, Gray RJ. A proportional hazards model for the subdistribution of a competing risk. J Am Stat Assoc. 1999;94(446):496-509. doi:10.1080/01621459.1999.10474144

[29] Vestergaard P, Mosekilde L. Fractures in patients with primary hyperparathyroidism: nationwide follow-up study of 1201 patients. World J Surg. 2003;27(3):343-349. doi:10.1007/s00268-002-6589-9 12607064

[30] Cummings SR, Melton LJ. Epidemiology and outcomes of osteoporotic fractures. Lancet. 2002;359(9319):1761-1767. doi:10.1016/S0140-6736(02)08657-9 12049882

[31] Hansen S, Hauge EM, Rasmussen L, Jensen JE, Brixen K. Parathyroidectomy improves bone geometry and microarchitecture in female patients with primary hyperparathyroidism: a one-year prospective controlled study using high-resolution peripheral quantitative computed tomography. J Bone Miner Res. 2012;27(5):1150-1158. doi:10.1002/jbmr.1540 22228118

[32] Anagnostis P, Vaitsi K, Veneti S, . Efficacy of parathyroidectomy compared with active surveillance in patients with mild asymptomatic primary hyperparathyroidism: a systematic review and meta-analysis of randomized-controlled studies. J Endocrinol Invest. 2021;44(6):1127-1137. doi:10.1007/s40618-020-01447-7 33074457

[33] Yeh MW, Zhou H, Adams AL, . The relationship of parathyroidectomy and bisphosphonates with fracture risk in primary hyperparathyroidism: an observational study. Ann Intern Med. 2016;164(11):715-723. doi:10.7326/M15-1232 27043778

[34] Seib CD, Meng T, Suh I, . Risk of fracture among older adults with primary hyperparathyroidism receiving parathyroidectomy vs nonoperative management. JAMA Intern Med. 2022;182(1):10-18. doi:10.1001/jamainternmed.2021.6437 34842909 PMC8630642

[35] Hedbäck G, Odén A. Increased risk of death from primary hyperparathyroidism—an update. Eur J Clin Invest. 1998;28(4):271-276. doi:10.1046/j.1365-2362.1998.00289.x 9615902

[36] Palmér M, Adami HO, Bergström R, Akerström G, Ljunghall S. Mortality after surgery for primary hyperparathyroidism: a follow-up of 441 patients operated on from 1956 to 1979. Surgery. 1987;102(1):1-7. 3589970

[37] Nilsson IL, Yin L, Lundgren E, Rastad J, Ekbom A. Clinical presentation of primary hyperparathyroidism in Europe—nationwide cohort analysis on mortality from nonmalignant causes. J Bone Miner Res. 2002;17(suppl 2):N68-N74. 12412780

[38] Vestergaard P, Mollerup CL, Frøkjaer VG, Christiansen P, Blichert-Toft M, Mosekilde L. Cardiovascular events before and after surgery for primary hyperparathyroidism. World J Surg. 2003;27(2):216-222. doi:10.1007/s00268-002-6541-z 12616440

[39] Tieland M, Trouwborst I, Clark BC. Skeletal muscle performance and ageing. J Cachexia Sarcopenia Muscle. 2018;9(1):3-19. doi:10.1002/jcsm.12238 29151281 PMC5803609

[40] Guirguis-Blake JM, Michael YL, Perdue LA, Coppola EL, Beil TL. Interventions to prevent falls in older adults: updated evidence report and systematic review for the US Preventive Services Task Force. JAMA. 2018;319(16):1705-1716. doi:10.1001/jama.2017.21962 29710140

