# Supplementary material for: Analysis of Comorbidities, Clinical Outcomes, and Parathyroidectomy in Adults With Primary Hyperparathyroidism
Source: JAMA Netw Open. 2022 Jun 3;5(6):e2215396. doi: 10.1001/jamanetworkopen.2022.15396 (PMC9166253; doi:10.1001/jamanetworkopen.2022.15396)
Supplement: Supplement. — eTable 1. Definitions eTable 2. Other Fracture Outcomes eTable 3. Cardiovascular Outcomes eTable 4. Overall Death and Cardiovascular Deaths eTable 5. Clinical Outcomes per Sex eTable 6. Clinical Outcomes Within One Year eFigure. Cumulative Incidence Function in PHPT Patients vs. Controls eTable 7. Subhazard Ratios for PHPT Patients vs Population Controls, With Consideration of Competing Risk of Death eTable 8. Outcomes for PTX Patients After Surgery Using the Same PTX Patients Before Surgery as Controls [file jamanetwopen-e2215396-s001.pdf]

Supplemental Online Content

Axelsson KF, Wallander M, Johansson H, et al. Analysis of comorbidities, clinical outcomes, and parathyroidectomy in adults with primary hyperparathyroidism. *JAMA Netw Open*. 2022;5(6):e2215396. doi:10.1001/jamanetworkopen.2022.15396

eTable 1. Definitions

eTable 2. Other Fracture Outcomes

eTable 3. Cardiovascular Outcomes

eTable 4. Overall Death and Cardiovascular Deaths

eTable 5. Clinical Outcomes per Sex

eTable 6. Clinical Outcomes Within One Year

eFigure. Cumulative Incidence Function in PHPT Patients vs. Controls

eTable 7. Subhazard Ratios for PHPT Patients vs. Population Controls, With Consideration of Competing Risk of Death

eTable 8. Outcomes for PTX Patients After Surgery Using the Same PTX Patients Before Surgery as Controls

This supplemental material has been provided by the authors to give readers additional information about their work.

© 2022 Axelsson KF et al. *JAMA Network Open.*

Introduction

For classification of diseases, sign and symptoms, surgical procedures, we used digital records of ICD-10 i.e. the 10th revision of the International Statistical Classification of Diseases and Related Health Problems (ICD), a medical classification list by the World Health Organization (WHO).

For classification of medications, we used The Anatomical Therapeutic Chemical (ATC) Classification System. It is overseen by the World Health Organization Collaborating Centre for Drug Statistics Methodology (WHOCC).

© 2022 Axelsson KF et al. *JAMA Network Open.*

eTable 1. Definitions

Variable	Definitions using ICD-10 or ATC codes
Fracture	S02, S12, S22, S32, S42, S52, S62, S72, S82, S92, T02, T08, T10, T12, T142, M485
Non-skeletal fall injury	W00-W19 code and a S00-T14 diagnosis, but not a simultaneous fracture
Cardiovascular event	I21-I22, I61, I63
Myocardial infarction	I21-I22
Ischemic stroke	I61
Hemorrhagic stroke	I63
Kidney stone	N20-N22
Charlson Comorbidity Component (weight)	
Dementia (1)	F00-F03
Ischeamic heart disease (1)	I20-I25
Heart failure (1)	I50
Cerebrovascular disease (1)	I60-I69
Vascular diseases (1)	I70-I79
Chronic pulmonary diseases (1)	J43-J46
Chronic liver disease (1)	K70-K77
Tumor without metastasis (2)	C00-C76, C80, C97
Lymphoma or leukemia (2)	C81-C96
Diabetes (1)	E10-E14
with end organ damage (+1)	E102, E103, E104, E105, E107, E112, E113, E114, E115, E117, H360
Kidney disease, renal failure (1)	N17-N19
moderate or severe (+1)	N18.2-N18.5
Hemiplegia (2)	G81
Peptic ulcer disease (2)	K25-K27
Metastatic solid tumor (6)	C77-C79
Osteoporosis	M80-M81
Alcohol related diseases	Mental and behavioural disorders due to use of alcohol (F10), Degeneration of nervous system due to alcohol (G31.2), Alcoholic polyneuropathy (G62.1), Alcoholic myopathy (G72.1), Alcoholic cardiomyopathy (I42.6), Alcoholic gastritis (K29.2), Alcoholic liver disease (K70), Alcohol-induced acute pancreatitis (K85.2), Alcohol-induced chronic pancreatitis (K86.0), Toxic effect of alcohol (T51)
Rheumatoid arthritis	M05-M06
Hyperthyroidism	E05

© 2022 Axelsson KF et al. *JAMA Network Open.*

Prednisolone use	Any previous three-month period in which more than 450 mg prednisolon (ATC-code H02AB06) were collected, i.e. more than 5 mg/day within a three-month period
Osteoporosis medication	Prescriptions: Bisphosphonates (M05BA and M05BB), Denosumab (M05BX04), Teriparatid (H05AA02), Strontium (M05BX03), Raloxifene (G03XC01), Testosterone (G03BA03), Systemic estrogens (G03CA) Tibolon (G03CX01).<br>Non-prescribed parenteral treatment offered to patients at outpatient clinics or while admitted: The combination of an osteoporosis diagnosis (M80, M81 or M859) and a code for intravenous (DT016) or subcutaneous (DT021) administration was used.
**Other medications:**	Repeated (≥2 collected prescriptions last year) and Recent (last prescription within 120 days)
Calcium and vitamin D	A12AX
Opioids	N02A
Antiepileptics	N03A
Anti-Parkinson drugs	N04
Antidepressants	N06A
Anti-dementia drugs	N06D
Thiazid diutetics	C03A
Beta-blockers	C07
Calcium antagonist	C08
RAS-inhibitors	C09

© 2022 Axelsson KF et al. *JAMA Network Open.*

eTable 2. Other Fracture Outcomes

		**Controls** N=163,740	**PHPT** N=16,374	**p-value**
	Time at risk, years, median (IQR)	4.62 (2.08-7.51)	1.15 (0.40-4.06)	
**Lower leg fracture**	Events, n (%)	2,544 (1.6%)	158 (1.0%)	
	per 1000 person-years (95% CI)	3.52 (3.38-3.66)	4.58 (3.89-5.35)	
	Cox, unadjusted, HR (95%CI)	Ref [1]	1.31 (1.12-1.54)	<0.001
	Cox, adjusted, HR (95%CI)	Ref [1]	1.19 (1.01-1.40)	0.03
**Upper arm fracture**	Events, n (%)	2,406 (1.5%)	167 (1.0%)	
	per 1000 person-years (95% CI)	3.32 (3.19-3.46)	4.84 (4.14-5.64)	
	Cox, unadjusted, HR (95%CI)	Ref [1]	1.46 (1.25-1.71)	<0.001
	Cox, adjusted, HR (95%CI)	Ref [1]	1.29 (1.10-1.51)	0.002
**Wrist fracture**	Events, n (%)	3,885 (2.4%)	248 (1.5%)	
	per 1000 person-years (95% CI)	5.40 (5.23-5.57)	7.24 (6.37-8.20)	
	Cox, unadjusted, HR (95%CI)	Ref [1]	1.34 (1.18-1.52)	<0.001
	Cox, adjusted, HR (95%CI)	Ref [1]	1.25 (1.10-1.42)	<0.001

Lower Leg Fracture (S82), Upper Arm Fracture (S422) and Wrist Fracture (S525-S526).
Adjusted Cox models adjusted for age, sex, Charlson comorbidity index and previous fracture.

© 2022 Axelsson KF et al. *JAMA Network Open.*

eTable 3. Cardiovascular Outcomes

	Follow-up truncated after 1 year		Maximum follow-up	
	**Controls** N=163,740	**PHPT** N=16,374	**Controls** N=163,740	**PHPT** N=16,374
Time at risk, years, median (IQR)	1.00 (1.00-1.00)	1.00 (0.40-1.00)	4.62 (2.08-7.51)	1.15 (0.40-4.06)
**Any cardiovascular event**				
Events, n (%)	1,735 (1.1%)	208 (1.3%)	9,350 (5.7%)	703 (4.3%)
per 1000 person-years (95% CI)	11.4 (10.8-11.9)	17.6 (15.3-20.2)	12.0 (11.7-12.2)	17.2 (16.0-18.5)
Cox, unadjusted, HR (95%CI)	Ref [1]	1.56 (1.35-1.80)*	Ref [1]	1.45 (1.34-1.57)*
Cox, adjusted, HR (95%CI)	Ref [1]	1.41 (1.22-1.63)*	Ref [1]	1.22 (1.13-1.32)*
**Acute myocardial infarction**				
Events, n (%)	797 (0.5%)	91 (0.6%)	4,346 (2.7%)	315 (1.9%)
per 1000 person-years (95% CI)	5.21 (4.85-5.58)	7.68 (6.18-9.42)	5.48 (5.32-5.65)	7.56 (6.75-8.45)
Cox, unadjusted, HR (95%CI)	Ref [1]	1.48 (1.19-1.84)*	Ref [1]	1.39 (1.24-1.56)*
Cox, adjusted, HR (95%CI)	Ref [1]	1.34 (1.08-1.67)**	Ref [1]	1.18 (1.05-1.32)**
**Hemorrhagic stroke**				
Events, n (%)	146 (0.1%)	15 (0.1%)	884 (0.5%)	50 (0.3%)
per 1000 person-years (95% CI)	0.95 (0.80-1.12)	1.26 (0.71-2.08)	1.10 (1.03-1.18)	1.18 (0.88-1.56)
Cox, unadjusted, HR (95%CI)	Ref [1]	1.32 (0.78-2.25)	Ref [1]	1.09 (0.82-1.45)
Cox, adjusted, HR (95%CI)	Ref [1]	1.19 (0.70-2.03)	Ref [1]	0.94 (0.71-1.26)
**Ischemic stroke**				
Events, n (%)	836 (0.5%)	109 (0.7%)	4,723 (2.9%)	370 (2.3%)
per 1000 person-years (95% CI)	5.46 (5.10-5.84)	9.21 (7.56-11.1)	5.96 (5.80-6.14)	8.91 (8.02-9.86)
Cox, unadjusted, HR (95%CI)	Ref [1]	1.70 (1.39-2.07)*	Ref [1]	1.51 (1.36-1.68)*
Cox, adjusted, HR (95%CI)	Ref [1]	1.54 (1.26-1.88)*	Ref [1]	1.25 (1.13-1.39)*

Any Cardiovascular Event (CVE) included admissions with myocardial infarction (I21-I22), hemorrhagic stroke (I63) or ischemic stroke (I61) with a seven-year washout period used for each diagnosis.
Adjusted Cox models were adjusted for age, sex, Charlson comorbidity index and previous CVE.
* p<0.001
** p<0.01

© 2022 Axelsson KF et al. *JAMA Network Open.*

eTable 4. Overall Death and Cardiovascular Deaths

	Follow-up truncated after 1 year		Maximum follow-up	
	**Controls** N=163,740	**PHPT** N=16,374	**Controls** N=163,740	**PHPT** N=16,374
Time at risk, years, median (IQR)	1.00 (1.00-1.00)	1.00 (0.40-1.00)	4.62 (2.08-7.51)	1.15 (0.40-4.06)
**Deaths overall**				
Events, n (%)	4,040 (2.5%)	580 (3.5%)	24,869 (15.2%)	2,193 (13.4%)
per 1000 person-years (95% CI)	26.3 (25.5-27.2)	48.8 (44.9-52.9)	31.0 (30.6-31.3)	51.8 (49.7-54.1)
Cox, unadjusted, HR (95%CI)	Ref [1]	1.85 (1.70-2.02)	Ref [1]	1.72 (1.65-1.80)
Cox, adjusted[a], HR (95%CI)	Ref [1]	1.49 (1.37-1.63)	Ref [1]	1.27 (1.22-1.33)
**Deaths related to cardiovascular disease**				
Events, n (%)	1,356 (0.8%)	210 (1.3%)	8,106 (5.0%)	722 (4.4%)
per 1000 person-years (95% CI)	8.84 (8.37-9.32)	17.7 (15.4-20.2)	10.1 (9.87-10.3)	17.1 (15.8-18.4)
Cox, unadjusted, HR (95%CI)	Ref [1]	2.00 (1.73-2.32)	Ref [1]	1.73 (1.60-1.86)
Cox, adjusted[b], HR (95%CI)	Ref [1]	1.62 (1.40-1.87)	Ref [1]	1.24 (1.15-1.34)
**Deaths related to ischemic heart disease (I20-I25)**				
Events, n (%)	829 (0.5%)	130 (0.8%)	4,903 (3.0%)	454 (2.8%)
per 1000 person-years (95% CI)	5.40 (5.04-5.78)	10.9 (9.14-13.0)	6.10 (5.93-6.28)	10.7 (9.77-11.8)
Cox, unadjusted, HR (95%CI)	Ref [1]	2.02 (1.68-2.43)	Ref [1]	1.79 (1.63-1.97)
Cox, adjusted[b], HR (95%CI)	Ref [1]	1.62 (1.35-1.95)	Ref [1]	1.27 (1.15-1.40)
**Deaths related to cerebrovascular disease (I60-I69)**				
Events, n (%)	639 (0.4%)	94 (0.6%)	3,927 (2.4%)	339 (2.1%)
per 1000 person-years (95% CI)	4.16 (3.85-4.50)	7.91 (6.39-9.68)	4.89 (4.74-5.04)	8.01 (7.18-8.91)
Cox, unadjusted, HR (95%CI)	Ref [1]	1.92 (1.55-2.38)	Ref [1]	1.68 (1.51-1.88)
Cox, adjusted[b], HR (95%CI)	Ref [1]	1.54 (1.24-1.91)	Ref [1]	1.20 (1.07-1.34)[c]

[a] Adjustment for age, sex, Charlson comorbidity index. [b] Adjustment for age, sex, Charlson comorbidity index and previous cardiovascular event. [c] p=0.002, for all other HR p<0.001. Deaths related to cardiovascular disease included deaths related to ischemic heart disease and cerebrovascular disease.

© 2022 Axelsson KF et al. *JAMA Network Open.*

eTable 5. Clinical Outcomes per Sex

	Men		Women	
	**Controls**	**PHPT**	**Controls**	**PHPT**
	N=35,680	N=3,568	N=128,060	N=12,806
Time at risk, years, median (IQR)	4.42 (1.97-7.44)	1.01 (0.36-3.66)	4.67 (2.12-7.52)	1.21 (0.42-4.18)
**Any fracture**				
Events, n (%)	2,586 (7.2%)	181 (5.1%)	14,740 (11.5%)	969 (7.6%)
per 1000 person-years (95% CI)	16.1 (15.5-16.7)	24.2 (20.8-28.0)	28.2 (27.8-28.7)	38.9 (36.5-41.5)
Cox, unadjusted, HR (95%CI)	Ref [1]	1.49 (1.28-1.73)	Ref [1]	1.37 (1.28-1.46)
Cox, adjusted, HR (95%CI)	Ref [1]	1.31 (1.12-1.52)	Ref [1]	1.21 (1.13-1.29)
**Hip fracture**				
Events, n (%)	679 (1.9%)	59 (1.7%)	3,840 (3.0%)	260 (2.0%)
per 1000 person-years (95% CI)	4.08 (3.78-4.40)	7.61 (5.79-9.82)	6.92 (6.70-7.14)	9.80 (8.64-11.1)
Cox, unadjusted, HR (95%CI)	Ref [1]	1.91 (1.46-2.49)	Ref [1]	1.44 (1.27-1.64)
Cox, adjusted, HR (95%CI)	Ref [1]	1.65 (1.26-2.15)	Ref [1]	1.13 (1.00-1.28)[a]
**Injurious fall**				
Events, n (%)	2,858 (8.0%)	214 (6.0%)	11,578 (9.0%)	916 (7.2%)
per 1000 person-years (95% CI)	17.5 (16.9-18.2)	26.5 (23.1-30.3)	19.4 (19.0-19.7)	29.1 (27.3-31.1)
Cox, unadjusted, HR (95%CI)	Ref [1]	1.50 (1.31-1.73)	Ref [1]	1.51 (1.41-1.61)
Cox, adjusted, HR (95%CI)	Ref [1]	1.32 (1.14-1.51)	Ref [1]	1.30 (1.21-1.39)
**Any cardiovascular event**				
Events, n (%)	2,569 (7.2%)	166 (4.7%)	6,781 (5.3%)	537 (4.2%)
per 1000 person-years (95% CI)	15.6 (15.0-16.2)	20.2 (17.2-23.5)	11.0 (10.8-11.3)	16.5 (15.1-17.9)
Cox, unadjusted, HR (95%CI)	Ref [1]	1.31 (1.12-1.53)	Ref [1]	1.51 (1.38-1.65)
Cox, adjusted, HR (95%CI)	Ref [1]	1.15 (0.99-1.35)[b]	Ref [1]	1.24 (1.13-1.35)
**Kidney stone**				
Events, n (%)	866 (2.4%)	156 (4.4%)	1,101 (0.9%)	228 (1.8%)
per 1000 person-years (95% CI)	5.14 (4.80-5.49)	19.1 (16.2-22.3)	1.75 (1.65-1.86)	6.86 (6.00-7.81)
Cox, unadjusted, HR (95%CI)	Ref [1]	3.59 (3.03-4.26)	Ref [1]	3.83 (3.32-4.42)
Cox, adjusted, HR (95%CI)	Ref [1]	2.54 (2.13-3.04)	Ref [1]	2.64 (2.27-3.07)
**Death**				
Events, n (%)	5,884 (16.5%)	511 (14.3%)	18,985 (14.8%)	1,682 (13.1%)
per 1000 person-years (95% CI)	34.4 (33.5-35.2)	59.7 (54.6-65.1)	30.0 (29.6-30.5)	49.8 (47.5-52.3)
Cox, unadjusted, HR (95%CI)	Ref [1]	1.77 (1.62-1.94)	Ref [1]	1.71 (1.63-1.80)
Cox, adjusted, HR (95%CI)	Ref [1]	1.35 (1.23-1.48)	Ref [1]	1.25 (1.19-1.31)
**Cardiovascular related deaths**				
Events, n (%)	2,153 (6.0%)	187 (5.2%)	5,953 (4.6%)	535 (4.2%)
per 1000 person-years (95% CI)	12.6 (12.0-13.1)	21.8 (18.8-25.2)	9.42 (9.18-9.66)	15.9 (14.5-17.3)
Cox, unadjusted, HR (95%CI)	Ref [1]	1.76 (1.52-2.05)	Ref [1]	1.72 (1.58-1.88)
Cox, adjusted, HR (95%CI)	Ref [1]	1.31 (1.12-1.52)	Ref [1]	1.21 (1.11-1.33)

Adjusted Cox models adjusted for age, sex, Charlson comorbidity index and the outcome in question (previous fracture, injurious fall, CVE or kidney stone respectively). All HR p-values <0.001 exept for a (p=0.05) and b (p = 0.08).

© 2022 Axelsson KF et al. *JAMA Network Open.*

eTable 6. Clinical Outcomes Within One Year

	Follow-up truncated after 1 year		Maximum follow-up	
	**Controls** N=163,740	**PHPT** N=16,374	**Controls** N=163,740	**PHPT** N=16,374
Time at risk, years, median (IQR)	1.00 (1.00-1.00)	1.00 (0.40-1.00)	4.62 (2.08-7.51)	1.15 (0.40-4.06)
**Any fracture**				
Events, n (%)	3,724 (2.3%)	417 (2.5%)	17,326 (10.6%)	1,150 (7.0%)
per 1000 person-years (95% CI)	25.8 (25.0-26.7)	39.8 (36.0-43.8)	25.4 (25.0-25.7)	35.5 (33.5-37.6)
Cox, unadjusted, HR (95%CI)	Ref [1]	1.53 (1.38-1.69)	Ref [1]	1.39 (1.31-1.48)
Cox, adjusted, HR (95%CI)	Ref [1]	1.38 (1.25-1.53)	Ref [1]	1.22 (1.15-1.30)
**Hip fracture**				
Events, n (%)	816 (0.5%)	90 (0.5%)	4,519 (2.8%)	319 (1.9%)
per 1000 person-years (95% CI)	5.61 (5.23-6.01)	8.47 (6.81-10.4)	6.26 (6.08-6.45)	9.30 (8.31-10.4)
Cox, unadjusted, HR (95%CI)	Ref [1]	1.51 (1.21-1.87)	Ref [1]	1.51 (1.35-1.70)
Cox, adjusted, HR (95%CI)	Ref [1]	1.34 (1.07-1.66[a]	Ref [1]	1.20 (1.07-1.35) [b]
**Injurious fall**				
Events, n (%)	2,904 (1.8%)	360 (2.2%)	14,436 (8.8%)	1,130 (6.9%)
per 1000 person-years (95% CI)	19.1 (18.4-19.8)	30.7 (27.6-34.1)	19.0 (18.7-19.3)	28.6 (27.0-30.3)
Cox, unadjusted, HR (95%CI)	Ref [1]	1.61 (1.44-1.79)	Ref [1]	1.51 (1.42-1.60)
Cox, adjusted, HR (95%CI)	Ref [1]	1.43 (1.28-1.60)	Ref [1]	1.30 (1.22-1.38)
**Any cardiovascular event**				
Events, n (%)	1,735 (1.1%)	208 (1.3%)	9,350 (5.7%)	703 (4.3%)
per 1000 person-years (95% CI)	11.4 (10.8-11.9)	17.6 (15.3-20.2)	12.0 (11.7-12.2)	17.2 (16.0-18.5)
Cox, unadjusted, HR (95%CI)	Ref [1]	1.56 (1.35-1.80)	Ref [1]	1.45 (1.34-1.57)
Cox, adjusted, HR (95%CI)	Ref [1]	1.41 (1.22-1.63)	Ref [1]	1.22 (1.13-1.32)
**Kidney stone**				
Events, n (%)	409 (0.2%)	150 (0.9%)	1,967 (1.2%)	384 (2.3%)
per 1000 person-years (95% CI)	2.67 (2.42-2.94)	12.7 (10.7-14.9)	2.47 (2.36-2.58)	9.27 (8.37-10.3)
Cox, unadjusted, HR (95%CI)	Ref [1]	4.72 (3.92-5.70)	Ref [1]	3.65 (3.27-4.08)
Cox, adjusted, HR (95%CI)	Ref [1]	3.16 (2.59-3.85)	Ref [1]	2.60 (2.32-2.91)
**Death**				
Events, n (%)	4,040 (2.5%)	580 (3.5%)	24,869 (15.2%)	2,193 (13.4%)
per 1000 person-years (95% CI)	26.3 (25.5-27.2)	48.8 (44.9-52.9)	31.0 (30.6-31.3)	51.8 (49.7-54.1)
Cox, unadjusted, HR (95%CI)	Ref [1]	1.85 (1.70-2.02)	Ref [1]	1.72 (1.65-1.80)
Cox, adjusted, HR (95%CI)	Ref [1]	1.49 (1.37-1.63)	Ref [1]	1.27 (1.22-1.33)
**Cardiovascular related deaths**				
Events, n (%)	1,356 (0.8%)	210 (1.3%)	8,106 (5.0%)	722 (4.4%)
per 1000 person-years (95% CI)	8.84 (8.37-9.32)	17.7 (15.4-20.2)	10.1 (9.87-10.3)	17.1 (15.8-18.4)
Cox, unadjusted, HR (95%CI)	Ref [1]	2.00 (1.73-2.32)	Ref [1]	1.73 (1.60-1.86)
Cox, adjusted, HR (95%CI)	Ref [1]	1.62 (1.40-1.87)	Ref [1]	1.24 (1.15-1.34)

Maximum follow-up (=Table 2) included for comparison, presented in shaded gray. Adjusted Cox models adjusted for age, sex, Charlson comorbidity index and the outcome in question (previous fracture, injurious fall, CVE or kidney stone respectively). All HR p-values <0.001 exept for a (p=0.009) and b (p = 0.002).

© 2022 Axelsson KF et al. *JAMA Network Open.*

eFigure. Cumulative Incidence Function in PHPT Patients vs. Controls

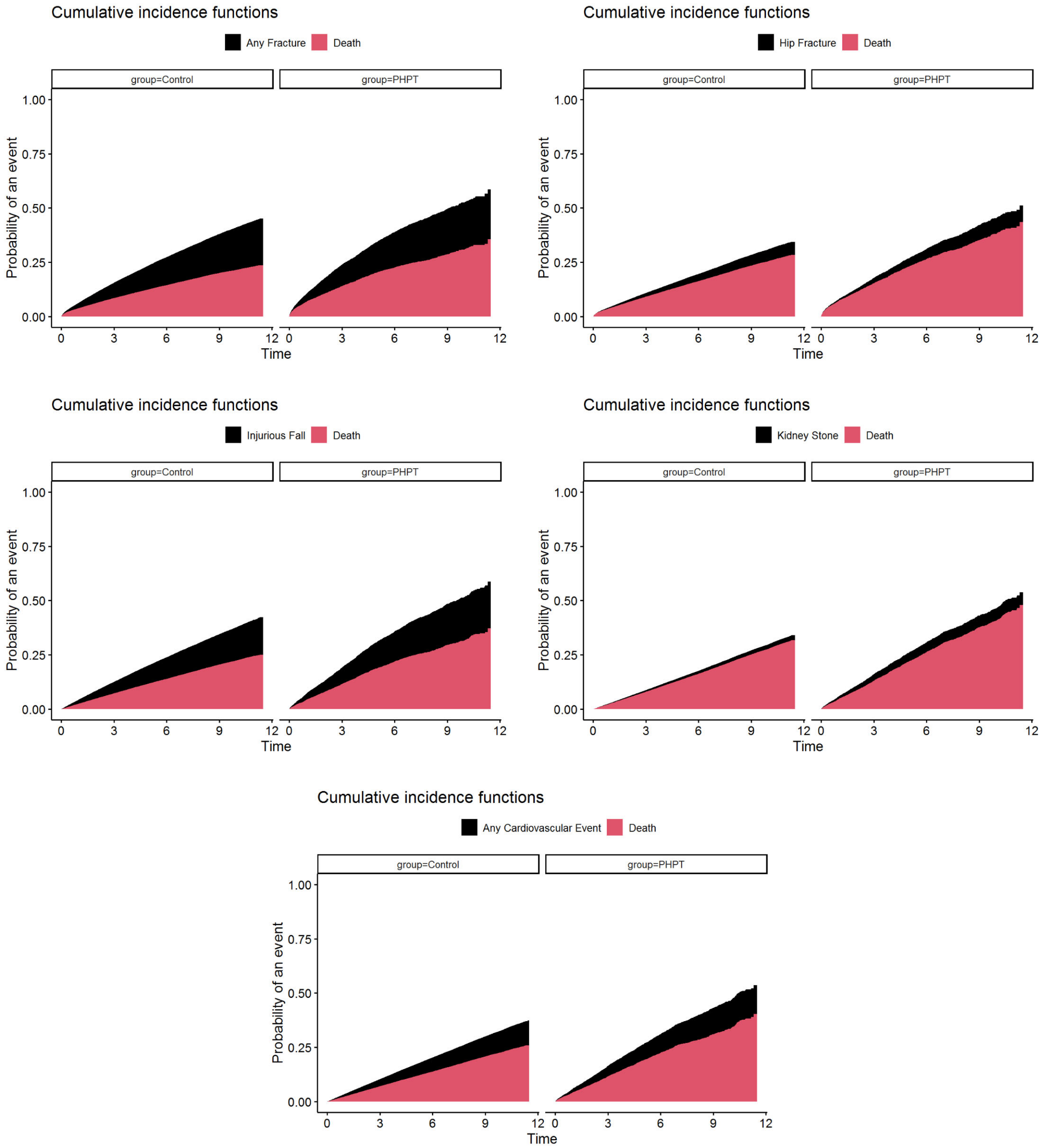

The cumulative incidence function, or subdistribution function, of fracture/injurious fall with death as competing risk was estimated using the Aalen-Johansen estimator. All patients and controls were included in the analysis.

© 2022 Axelsson KF et al. *JAMA Network Open.*

eTable 7. Subhazard Ratios for PHPT Patients vs. Population Controls, With Consideration of Competing Risk of Death

	Follow-up			
	Truncated After 1 year		Maximum	
Event	SHR (95%CI)	p-value	SHR (95%CI)	p-value
Any fracture	1.57 (1.36-1.81)	<0.001	1.22 (1.13-1.31)	<0.001
Major osteoporotic fracture	1.73 (1.45-2.07)	<0.001	1.27 (1.16-1.39)	<0.001
Hip fracture	1.67 (1.23-2.26)	0.001	1.37 (1.19-1.58)	<0.001
Injurious fall	1.53 (1.32-1.79)	<0.001	1.31 (1.22-1.42)	<0.001
Any cardiovascular Event	1.35 (1.11-1.64)	0.003	1.29 (1.17-1.42)	<0.001
Kidney stone	3.97 (2.87-5.50)	<0.001	3.22 (2.72-3.82)	<0.001

Subhazard ratios (SHR) with 95% CI for fractures, injurious falls, any cardiovascular events and kidney stones using the Fine & Grey model with death as the competing risk. The analyses was performed in a subset of 32,748 individuals, comparing all 16,374 PHPT patients and 16,374 controls matched according to age, sex and county of residence (=first control assigned per case in main analysis). Results are presented for the maximum available and for one-year follow-up.

© 2022 Axelsson KF et al. *JAMA Network Open.*

eTable 8. Outcomes for PTX Patients After Surgery Using the Same PTX Patients Before Surgery as Controls

	PTX Patients		p-value
	**Pre PTX**	**Post PTX**	
	N=6,934	N=6,934	
Time at Risk, years, median (IQR)	0.48 (0.24-0.92)	4.78 (2.21-7.18)	
**Any fracture**			
Events, n (%)	139 (2.0%)	589 (8.5%)	
Per 1000 person-years (95% CI)	27.8 (23.4-32.9)	21.5 (19.8-23.3)	
Cox, adj for age and sex, HR (95% CI)	Ref [1]	0.77 (0.63-0.95)	0.01
Cox, outcome-specific adjustment*, HR (95% CI)	Ref [1]	0.76 (0.62-0.93)	0.008
**Major osteoporotic fracture**			
Events, n (%)	79 (1.1%)	362 (5.2%)	
Per 1000 person-years (95% CI)	15.6 (12.4-19.4)	12.9 (11.6-14.3)	
Cox, adj for age and sex, HR (95% CI)	Ref [1]	0.75 (0.57-0.99)	0.04
Cox, outcome-specific adjustment*, HR (95% CI)	Ref [1]	0.74 (0.56-0.97)	0.03
**Injurious fall**			
Events, n (%)	90 (1.3%)	577 (8.3%)	
Per 1000 person-years (95% CI)	16.1 (13.0-19.8)	18.1 (16.7-19.6)	
Cox, adj for age and sex, HR (95% CI)	Ref [1]	1.02 (0.80-1.31)	0.85
Cox, outcome-specific adjustment*, HR (95% CI)	Ref [1]	1.01 (0.79-1.30)	0.91
**Kidney stone**			
Events, n (%)	112 (1.6%)	277 (4.0%)	
Per 1000 person-years (95% CI)	20.0 (16.5-24.1)	8.48 (7.51-9.54)	
Cox, adj for age and sex, HR (95% CI)	Ref [1]	0.61 (0.48-0.78)	<0.001
Cox, outcome-specific adjustment*, HR (95% CI)	Ref [1]	0.54 (0.42-0.69)	<0.001

PTX patients were followed from PHPT diagnosis to the date of PTX (PrePTX) and from date of PTX to end of study, emigration or death (PostPTX). Cox proportional hazards models were used to study the risk of outcomes before (reference) and after PTX. *Adjustment for age, sex, Charlson Comorbidity Index and the investigated outcome (previous fracture, injurious fall, CVE or kidney stone respectively). Only outcomes with at least 30 events in the PrePTX period were analysed.

© 2022 Axelsson KF et al. *JAMA Network Open.*
